# Is Dietary Milk Intake Associated with Cataract Extraction History in Older Adults? An Analysis from the US Population

**DOI:** 10.1155/2020/2562875

**Published:** 2020-02-19

**Authors:** Osama M. Mustafa, Yassine J. Daoud

**Affiliations:** Cornea, Cataract, and Refractive Surgery Division, The Johns Hopkins University Wilmer Eye Institute, Baltimore, MD 21287, USA

## Abstract

**Background:**

Galactose accumulation in the lens tissue is known to be cataractogenic. Whether consistent dietary intake of lactose—which consists of glucose and galactose—predisposes to senile cataract remains unclear. This study was conducted to investigate the association between a number of dietary milk intake indicators and cataract extraction history in a representative sample of older adults from the US population. *Methods and Materials*. This is a cross-sectional, population-based study. Participants of the United States National Health and Nutrition Examination Survey 2001–2008 who were ≥50 years old and provided a complete history of their usual daily dietary intake were included. Exclusion criteria were special diets, extreme daily energy intake, and missing outcome (i.e., cataract extraction history). Indicators of milk intake used were early-life intake regularity, current daily milk/total dairy intake amounts, and estimated lifelong milk exposure. Odds ratios (OR) and 99% confidence intervals (99% CI) were calculated with fitting weights to better represent the population-based estimates.

**Results:**

Among the 5930 studied participants, early-life milk intake regularity was not associated with cataract extraction history in age/sex/ethnicity-adjusted and multivariable-adjusted models (*p* trend = 0.064 and 0.094, respectively). Current daily milk intake was associated with a slight reduction in the likelihood of cataract extraction in the age/sex/ethnicity-adjusted model (OR = 0.885 per cup equivalents, 99% CI = 0.795–0.986) and in the multivariable model (OR = 0.871 per cup equivalents, 99% CI = 0.746–0.993). However, no such association was observed between quartiles of current dietary milk intake and cataract extraction history (*p* trend = 0.064 and 0.094, respectively). Current daily milk intake was associated with a slight reduction in the likelihood of cataract extraction in the age/sex/ethnicity-adjusted model (OR = 0.885 per cup equivalents, 99% CI = 0.795–0.986) and in the multivariable model (OR = 0.871 per cup equivalents, 99% CI = 0.746–0.993). However, no such association was observed between quartiles of current dietary milk intake and cataract extraction history (*p* trend = 0.064 and 0.094, respectively). Current daily milk intake was associated with a slight reduction in the likelihood of cataract extraction in the age/sex/ethnicity-adjusted model (OR = 0.885 per cup equivalents, 99% CI = 0.795–0.986) and in the multivariable model (OR = 0.871 per cup equivalents, 99% CI = 0.746–0.993). However, no such association was observed between quartiles of current dietary milk intake and cataract extraction history (

**Conclusion:**

There appears to be no direct relationship between several indicators of dietary milk consumption and cataract extraction history in the general American population.

## 1. Introduction

Several mechanisms, such as oxidative and osmotic stress, mediate cataract development. In states of oxidative stress, the production of free radicals exceeds the tissue's elimination capacity, resulting in the deposition of alpha-crystallin and impairment of lens transparency. In osmotic stress states, excess sugars (e.g., galactose) are converted to their sugar alcohol counterparts (e.g., galactitol), the accumulation of which disturbs water and electrolyte homeostatic mechanisms necessary to maintain tissue transparency [1]. This osmotic imbalance is believed to underlie the early development of cataract in inborn disorders of galactose metabolism, such as galactokinase deficiency and galactosemia [2]. In addition to the direct osmotic damage caused by its accumulation, recent evidence suggests that galactose could accelerate lens epithelial cell senescence *in vitro*, possibly by impairing autophagy and mitochondrial function [3]. The accumulation of sugar alcohols is also believed to play a role in hyperglycemia- and diabetes-induced cataracts [4].

Because of the modulatory role of diet in disease outcomes, and the cataractogenic potential of galactose, researchers speculated that long-term dietary intake of galactose might be associated with senile cataract formation [5]. In human diet, the major source of galactose is lactose in milk and dairy products. To date, the effect of dietary milk consumption on cataract development remains controversial. Early reports from regions of Asia concluded that possible links exist [5, 6]. However, recent investigations in European and Mediterranean populations were less conclusive [7, 8]. In this study, we examined the relationship between indicators of dietary milk exposure and cataract extraction history in a nationally representative sample of the American population.

## 2. Materials and Methods

### 2.1. Study Design and Population

This is a cross-sectional analysis of nationally representative data of adults, aged 50 years and over, participating in the National Health and Nutrition Examination Survey (NHANES). The protocols were approved by the National Center for Health Statistics Research Ethics Review Board and conducted in line with the tenets of the Declaration of Helsinki. Participants who provided a complete first-day dietary survey from 2001 through 2008 (*n* = 37521) were eligible for inclusion in this analysis if they were ≥50 years old and considered the detailed 24 h food intake as representative of their usual intake (*n* = 6904). Eligible participants on special diets (*n* = 927), with extreme daily total energy intake (≥3 standard deviations above or below the log-transformed sample mean) [9] (*n* = 44), and/or additionally missing the outcome variable (*n* = 3) were excluded. Therefore, a total of 5930 participants were included in this analysis.

### 2.2. Milk Intake Assessment and Indicators

Early-life daily milk intake regularity was reported for three age intervals: 5–12, 13–17, and 18–35 years of age. Participants reporting milk drinking “once a day or more” during *all* three intervals were considered to be always-regular daily milk drinkers from childhood through early adulthood. Current daily milk intake (and total dairy intake) was assessed using 24-hour dietary recall using United States Department of Agriculture (USDA) automated multiple-pass method [10]. The servings (in cup equivalents) were determined using MyPyramid Equivalent Databases and Food Patterns Equivalent Databases relevant to each of the NHANES cycles [11]. The participant-specific amounts of current daily milk intake and total dairy intake were assessed on a continuous scale as well as in sample-based quartiles. Self-perceived consistency of milk intake was identified by asking the participants whether they had been drinking milk at least 5 times a week for most/all of their life.

Since tracing lifelong exposure to a particular dietary component often proves difficult, the attempt was made to evaluate the lifelong/long-term milk exposure using a proxy indicator that categorizes participants based on combinations of consistency and amounts of early-life and current milk intake, respectively ([Supplementary-material supplementary-material-1], Supplementary Materials). The high exposure group was defined by participants who were always-regular early-life daily milk drinkers and had highest quartile of current milk intake, while the low exposure group consisted of the participants who were never regular early-life milk drinkers and were of the lowest current milk intake quartile group. The categorization of remaining groups is shown in [Supplementary-material supplementary-material-1].

### 2.3. Outcome and Confounding Factors

The outcome of interest in this study was cataract extraction history. To control for demographic and lifestyle variables that may confound the relationship between the examined indicators and the outcome, we adjusted for age, sex, and ethnicity in the baseline models. In addition, educational attainment, income [12], health insurance status, smoking status, alcohol drinking history, body mass index (BMI), diabetes history, and hypertension history were adjusted for in the multivariable models. A positive history of diabetes was identified by the self-report of physician-diagnosed disease and/or by taking diabetic medications/insulin at the time of survey. Hypertension was defined by self-report of diagnosis by a physician or other health professional at two different visits or by taking antihypertensive medications at the time of survey. BMI was calculated as the weight by height (kg/m^2^). Furthermore, total energy intake (kcal) was controlled for in analyses that included current dietary milk and total dairy intakes, including lifelong milk exposure.

### 2.4. Statistical Analysis

Characteristics of the study sample and milk consumption indicators were summarized as weighted means and percentages for continuous and categorical variables, respectively. The relationship between milk-based predictors and outcome was initially explored univariably as well as with adjustments of age, sex, and ethnicity. Then, potential confounding factors were collectively controlled for to produce the final multiple logistic regression models. The analysis was conducted using SPSS CS v.22 (IBM Corp, Armonk, NY). Odds ratios and 99% confidence intervals (99% CI) were calculated and the corresponding alpha error rate of <0.01 (two-tailed) was considered for statistical significance. To better reflect the US national population estimates, the data were analyzed using appropriate weights to account for survey nonresponse, group oversampling, and dietary intake variation associated with the complex structure of the survey [13].

## 3. Results

The sociodemographic characteristics of the study population are shown in Table 1. The mean age of participants was 63.8 ± 9.9 years, of whom 15.7% had a positive history of cataract extraction. On average, participants reported current daily consumption of 0.91 ± 1.14 and 1.43 ± 1.39 cup equivalents/day of milk and total dairy products, respectively, at the time of the survey (Table 2). About 17% and 8% were estimated to have had high and low lifelong exposure to milk, respectively.

### 3.1. Cataract and Early-Life Milk Consumption

When using daily (i.e., ≥7 times a week) milk drinking as an indication of regular milk drinking in early life, 44.5% of participants were considered regular daily milk drinkers throughout the childhood to early adulthood period (i.e., 5–35 years) (Table 2). Over a third (35.1%) of participants were less regularly drinking milk during one or more of the early-life intervals (i.e., 5–12, 13–17, and 18–35 years) and, thus, were considered to have a variable regular daily intake during the childhood to early adulthood period. The remaining 20.3%, who previously reported never being regular milk drinkers most or all of their life, were considered to have never had regular milk intake during childhood to early adulthood period. Neither univariable nor multivariable examination showed a statistically significant association between early-life milk intake and cataract extraction history (Table 3).

### 3.2. Cataract and Current Daily Milk Intake

The median number of cup equivalents of daily milk intake was 0.53 (IQR = 0.12–1.27) at the time of the survey. Univariable analysis did not show a significant association between current daily intake and cataract extraction history (OR = 0.970 per cup equivalent, 99% CI = 0.891–1.055; *p* = 0.336). In contrast, age/sex/ethnicity-adjusted and multivariable models suggested a reduction in the risk of cataract extraction history with the reported current milk intake (OR = 0.885 per cup equivalent, 99% CI = 0.795–0.986, *p* = 0.004 and OR = 0.871 per cup equivalent, 99% CI = 0.746–0.993, *p* = 0.007, respectively). However, when examining participants in quartiles of current daily milk intake, it was not found to be associated with cataract extraction history in the studied population (Table 3).

### 3.3. Cataract and Current Total Dairy Intake

The median number of cup equivalents of total dairy product intake was 1.07 (IQR = 0.44–2.02) at the time of the survey. Univariable examination suggested a statistically significant decrease in the odds of cataract extraction history with increased intake (OR = 0.891, 99% CI = 0.825–0.963, *p* < 0.001). However, this association was not found to be significant after adjusting for age and other confounding factors (age/gender/ethnicity-adjusted OR = 0.914, 99% CI = 0.815–1.026, *p* = 0.042 and multivariable-adjusted OR = 0.910, 99% CI = 0.798–1.039, *p* = 0.063, respectively). Likewise, examination of the relationship between quartiles of total dairy intake and cataract extraction history did not show any significant findings (Table 3).

### 3.4. Cataract and Lifelong Milk Exposure

No significant univariable association was observed between lifelong milk exposure and cataract extraction history (*p*=0.543). Moreover, although the model adjusting for age, gender, ethnicity, and total energy intake suggested a significant trend between lifelong milk exposure and cataract extraction history (*p*=0.002), none of the individual groups were significantly different from the high exposure group (*p* > 0.01, Table 3). Also, after adjusting for all potential confounders, neither the trend of association nor the individual groups met the criterion of significance in this study (Table 3).

### 3.5. Cataract and Self-Perceived Consistency of Milk Consumption

Approximately 45.4% perceived themselves as consistent milk drinkers (≥5 times/week) for most or all of their life, whereas 20.2% said that they never consumed milk consistently (Table 2). Univariate examination revealed no significant association between self-perceived intake consistency and cataract extraction history (OR = 1.088, 99% CI = 0.827–1.432 and OR = 0.956, 99% CI = 0.751–1.216 for the never-consistent and sometimes-consistent groups, respectively, compared to the consistent group; *p* trend = 0.476). After adjustment for age, gender, and ethnicity, the association was statistically significant (*p* trend = 0.004), with higher odds in participants who reported variable/sometimes-consistent milk drinkers compared to consistent milk drinkers (Table 3). However, this relationship was not found to be significant after controlling for potential confounding variables (Table 3).

## 4. Discussion

The potential role of galactose in cataract development was first identified in 1935 in rats that were fed a high-galactose diet [14]. A decade later, the observation of cataract development in children with inborn galactose metabolism errors was made [15]. Because of the known connection between galactose metabolism and cataractogenesis, researchers postulated that the long-term, high intake of milk products might cause subliminal but cumulative damage to the lens, leading to senile cataract formation [16]. Some even suggested decreasing dietary intake of milk and dairy products or using inhibitors of pertinent enzymes to treat predisposed individuals [17, 18]. In this study, we explored the association between several indicators of dietary milk consumption and cataract extraction history in older adults. To our knowledge, the relationship between life-long milk consumption and cataract has not been explored previously in a representative sample of the general US population.

While the link between galactose-mediated damage and congenital cataract in galactosemic patients is well-established, the evidence of a relationship between milk consumption and age-related cataract in normal individuals has been, at best, inconclusive. Earlier epidemiological studies suggested that a relationship might exist [5, 6, 19]. In one study, the geographic distribution of milk consumption and lactose malabsorption in relation to the incidence of cataract was used to imply a possible link [5]. In another, self-reported daily milk intake was compared between cases of cataract and consecutive healthy controls in ophthalmic clinic patients [6]. The authors found that higher milk intake was positively correlated with cortical cataracts [6].

However, more recent studies have found no such association between milk intake and cataract risk. For example, case-control studies did not find a significant difference in cataract [7] or cataract extraction risk [20] based on milk and dairy product consumption. Likewise, a recently published prospective study of adult Mediterranean subjects with high cardiovascular risk found no association between cataract incidence and total dairy products intake, including milk [8]. These findings are consistent with what we observed in this large, population-based study. We attempted to examine the possibility that individuals with consistently higher lactose levels accrue greater damage over long years of milk consumption [21] and found it to be insignificant. We also explored the possibility of such an effect being specifically related to consumption in the early years of a participant's life (i.e., 5–35 years) and similarly found no significant association.

A possible explanation for the lack of direct association is that hypergalactosemia alone may be insufficient to cause damage to the lens in healthy individuals. As some empirical evidence suggests, immediate susceptibility combined with high-galactose intake is needed to affect the lens. This notion is consistent with the synergistic effect observed when two cataractogenic factors are introduced simultaneously in an animal model [22]. For example, an investigation of the effect of high milk intake in Sprague-Dawley rats showed that the presence of underlying damage to the lens (e.g., by naphthalene) led to significant susceptibility to injury by excessive milk intake [23]. This notion is also in line with previous clinical observations suggesting that high lactose intake was related to cataract risk in subjects with low activity of galactose-metabolic enzymes, but not when high levels of enzymatic activity were present [24].

We also found no clear association between the quartiles of current daily milk or total dairy intake and cataract history, a finding which is similar to that reported in a case-control study examining a Greek sample [7]. Possibly, because the nutritional profile of milk is rich with various nutrients, some components may “neutralize” the cataractogenic effects of lactose/galactose. For example, in one experiment, coadministering whey along with purified lactose in rats inhibited the formation of cataract that was otherwise observed when fed purified lactose alone [25]. Moreover, consistent intake of milk may replace other less healthy dietary behaviors/patterns that could otherwise increase the risk of cataract [26].

Interestingly, we did find a significant reduction in cataract history risk when assessing milk intake on a continuous scale. While it is less often encountered in the literature, a protective effect of dairy is reported occasionally with a specific dairy product [8, 20] or, less commonly, for a specific cataract type [27]. In the recent study examining Mediterranean adults, skimmed yogurt intake (but not milk or other types of dairy products) was associated with reduced cataract risk [8]. The authors postulated that this protection could be mediated by reduction in cardiovascular risk factors, such as diabetes, or inflammation. Nonetheless, other dairy products, including milk, examined in the same populations failed to show any association with cataract risk [8, 20]. Hence, the possibility of the observed effect being coincidental cannot be ruled out.

As alluded to earlier, there are interesting discrepancies between findings from older vs. more recent reports regarding the association between milk intake and cataract. Multiple factors could have played a role. References to data stemming from animal experimentation were common [14, 23, 25], but these models required notably high intake of lactose (25–70% of total dietary intake) that is unlikely to be applicable to the human diet [5]. Besides, different indicators of milk exposure were historically used to assess this relationship in humans. For example, one approach was to examine expression of galactosemic enzymes in RBCs and correlate it with cataract [28, 29]. Another approach was geographic segregation of milk consumption, lactose malabsorption, and cataract prevalence [5]. In the latter approach, the concordance found in the segregation of regions of high milk consumption and lactose absorption with high cataract incidence may be appealing, but one must exercise caution to avoid assigning group-level indicators to individual-level outcomes (i.e., ecological bias) [30].

While each approach is bound to have some limitations, we herein examined a set of individual-level indicators of milk consumption to assess the outcome. Therefore, conclusions are not prone to ecological bias. In addition, previous research indicates that the individual-level approach, used in this study, is more resilient to model misspecification than ecological studies [31]. Other noteworthy strengths of this study are the inclusive, nationally representative sampling of the population and the use of dietary data collected through a standardized, prevalidated protocol [10]. Moreover, indicators used herein covered patterns of both current and past milk consumption in amount and frequency, respectively. The intent was to assess the relationship from multiple perspectives so that better conclusions could be reached.

Nonetheless, this study has some limitations. Because the milk-related indicators were self-reported, there is a potential for recall bias. In addition, indicators and outcomes were collected cross-sectionally, and thus, causality of any observed findings could not have been established. Another limitation is that the subtypes of cataracts in the studied participants were unknown. Previous reports suggested the presence of links between cortical cataract formation and osmotic stress, particularly at the initial stages [32]. On the other hand, this link seems to be less relevant to the age-related nuclear cataracts, which are largely oxidative stress-driven [32]. Therefore, a slight association of milk consumption with certain subtypes of cataract cannot be ruled out [6]. Also, the indicators of early-life consumption covered the regularity of milk consumption without the exact amounts. Finally, the focus of indicators used in this study was on the intake amounts and consistency. In conceptual terms, factors affecting serum lactose/galactose (and galactitol) levels would be the amount and consistency of milk consumption, intestinal lactase activity, and galactose absorption [33]. In turn, factors modulating intralenticular galactose levels (and cataract risk) include serum galactose levels, diffusion rate across aqueous humor and lens capsule, activity of galactose pathway enzymes [2, 24], and activity of enzymes of sugar alcohol metabolism within lens tissue [1, 2]. Therefore, from a holistic perspective, examining the sequence of factors in this pathway from lactose intake to intralenticular galactose levels would be necessary to better understand its relationship with cataract risk in the general population. While such investigation would be cost-prohibitive at the population level, further focused investigations that use this strategy are encouraged.

## 5. Conclusion

While the link may be more relevant to susceptible individuals, milk consumption does not appear to be associated with the development of age-related cataract in the general population.

## Figures and Tables

**Table 1:** Sociodemographic and baseline characteristics of the study population.

	Summary finding
Age (year), mean ± SD	63.8 ± 9.9

Sex (%)	
Male	49.0
Female	51.0

Race/ethnicity (%)	
Mexican American, other Hispanic, other race	11.5
Non-Hispanic white	80.4
Non-Hispanic black	8.2

Level of education (%)	
<9^th^ grade	9.7
9^th^–11^th^ grade	11.2
High school or equivalent	28.0
Some college education or AA degree	25.8
College graduate or higher	25.2

Income to poverty ratio (%)	
Below 100%	8.9
100%–300%	38.6
More than 300%	52.5

Current health insurance (%)	
Yes	91.5
No	8.5

Smoking status (%)	
Nonsmoker	47.3
Current or former smoker	52.7

Alcohol consumption history (%)	
Lifelong abstinent	14.2
No history of binge drinking	71.6
Positive history of binge drinking	14.2

Diabetes mellitus history (%)	
Positive	11.6
Negative	88.4

Hypertension history (%)	
Positive	43.8
Negative	56.2

Body mass index (kg/m^2^), %	
(Mean ± SD)	(28.3 ± 5.9)
Underweight and normal weight (<25)	30.0
Overweight (25 to <30)	38.8
Obese (≥30)	31.3

Had cataract extraction (%)	
Yes	15.7
No	84.3

**Table 2:** Summary statistics of milk consumption indicators, including usual daily intake amounts (in cup equivalents) of milk and total dairy products, and the estimated level of lifelong milk exposure by participants of 24 h dietary survey.

	Total	Positive cataract extraction history (% within-group)	Negative cataract extraction history (% within-group)	Univariate *p* value
Current total milk intake (cup equivalents/day), mean ± SD	0.91 ± 1.14	0.88 ± 0.95	0.92 ± 1.17	0.336

Current total dairy intake (cup equivalents/day), mean ± SD	1.43 ± 1.40	1.26 ± 1.13	1.46 ± 1.44	<0.001

Early-life regular daily milk drinking (%)				
Self-reported as never a regular milk drinker	20.3	22.0	20.10	
Variable (sometimes daily)	35.1	32.8	35.60	0.224
Always-regular daily milk drinker	44.5	45.2	44.40	

Estimated lifelong milk exposure (%)				
Low	7.9	6.5	8.1	0.543
Below average	20.3	21.2	20.2	
Average	29.3	29.2	29.3	
Above average	25.4	25.3	25.4	
High	17.1	17.8	17.0	

Self-perceived consistency (≥5/week) of milk drinking throughout life (%)				
Yes	45.4	45.3	45.4	0.476
Sometimes but not always	34.4	33.0	34.6	
Never	20.2	21.7	20.0	

**Table 3:** The odds ratio estimates of several indicators of milk intake in relation to cataract extraction history in a sample of ≥50-year-old participants of the National Health and Nutrition Examination Survey 2000–2008.

	Age, gender, and ethnicity-adjusted^*∗*^		Multivariable	
	OR (99%CI)	*p* value	OR (99% CI)	*p* value
Early-life daily milk consumption		*p*=0.064		*p*=0.094
Never regular daily milk drinker	1.250 (0.925–1.690)	0.053	1.332 (0.943–1.881)	0.031
Variable (sometimes daily)	1.240 (0.938–1.638)	0.045	1.158 (0.825–1.624)	0.255
Regular daily milk drinker	Reference	—	Reference	—

Current milk intake	*p*=0.154		*p*=0.317	
Q1	1.262 (0.918–1.734)	0.057	1.272 (0.893–1.812)	0.076
Q2	1.299 (0.856–1.971)	0.10	1.267 (0.799–2.009)	0.178
Q3	1.037 (0.764–1.406)	0.754	1.120 (0.797–1.575)	0.379
Q4	Reference	—	Reference	—

Current total dairy intake	*p*=0.836		*p*=0.528	
Q1	1.268 (0.891–1.804)	0.078	1.251 (0.910–1.72)	0.165
Q2	1.123 (0.769–1.642)	0.418	1.202 (0.863–1.674)	0.272
Q3	1.097 (0.801–1.503)	0.437	1.138 (0.890–1.454)	0.296
Q4	Reference	—	Reference	—

Estimated lifelong exposure		**p**=0.002		*p*=0.049
Low	1.019 (0.660–1.572)	0.911	1.239 (0.795–1.931)	0.203
Below average	1.396 (0.972–2.003)	0.017	1.340 (0.884–2.032)	0.066
Average	1.342 (0.940–1.916)	0.032	1.350 (0.924–1.974)	0.039
Above average	0.931 (0.663–1.307)	0.575	0.954 (0.664–1.372)*p*	0.734
High	Reference	—	Reference	—

Self-perceived consistency of milk consumption		**p**=0.004		*p*=0.018
Never been drinking milk ≥5 times/week	1.289 (0.957–1.736)	0.027	1.376 (0.980–1.933)	0.015
Sometimes but not always	1.359 (1.065–1.733)	**0.001**	1.252 (0.965–1.624)	0.025
Have been drinking milk ≥5 times/week for most or all of my life	Reference	—	Reference	—

OR, odds ratio; CI, confidence interval; *Q*, quartile. ^*∗*^Also adjusted for energy intake for variables containing current milk and total dairy intake. **Bold** indicates statistical significance (*p* < 0.01).

## Data Availability

The data generated and/or analyzed in the current study are available in the US National Center for Health Statistics repository.

## Supplementary Materials

Supplementary Materials Table S1: categorization of the composite lifelong milk exposure variable.
