# Supplementary material for: Is Dietary Milk Intake Associated with Cataract Extraction History in Older Adults? An Analysis from the US Population
Source: J Ophthalmol. 2020 Feb 19;2020:2562875. doi: 10.1155/2020/2562875 (PMC7053483; doi:10.1155/2020/2562875)
Supplement: Supplementary Materials — Table S1: categorization of the composite lifelong milk exposure variable. [file 2562875.f1.docx]

**Supplementary material:**

**Table S1.** Categories of the composite “lifelong milk exposure” variable in relation to early-life consistency of milk consumption and quartiles of current milk intake amounts.

|  | | **Current milk drinking** | | |
| --- | --- | --- | --- | --- |
|  |  | **Q1** | **Q2-Q3** | **Q4** |
| **Milk drinking during childhood to early-adulthood** | **Never regular** | Low lifelong exposure | Below average | Average lifelong exposure |
|  | **Variably regular** | Below average | Average lifelong exposure | Above average |
|  | **Always regular*** | Average lifelong exposure | Above average | High lifelong exposure |

Q1, first quartile; Q2-Q3, second and third quartile; Q4, fourth quartile

*At least daily during 5-35 years of age.
